# iKids study protocol: a longitudinal study to understand the impact of interactive electronic devices on the development and health of young children in England

**DOI:** 10.1136/bmjopen-2025-101523

**Published:** 2025-08-01

**Authors:** Liane Beretta De Azevedo, John Stephenson, Amy Hughes, Jenny Retzler, John J Reilly, Stuart Fairclough, Tony Okely, Daniel Jones, Christine Smith, João Paulo de Aguiar Greca, Colette Marr

**Affiliations:** 1 Sheffield Hallam University, Sheffield, UK; 2 Health Sciences, University of Huddersfield, Huddersfield, UK; 3 University of Leeds, Leeds, UK; 4 University of Strathclyde, Glasgow, UK; 5 Health Research Institute and Department of Sport and Physical Activity, Edge Hill University, Ormskirk, UK; 6 School of Education, University of Wollongong, Wollongong, New South Wales, Australia; 7 Teesside University, Middlesbrough, UK; 8 Christine Smith Consultancy, Huddersfield, UK; 9 Human Nutrition and Exercise Research Centre, Newcastle University Population Health Sciences Institute, Newcastle upon Tyne, UK

**Keywords:** Behavior, Child, Digital Technology, Observational Study, PUBLIC HEALTH

## Abstract

**Introduction:**

There is evidence of both positive and negative impacts of interactive electronic devices (IEDs), such as tablets and smartphones, on young children’s development and health outcomes. Consultations with early years practitioners, parents and policy makers recognise IEDs as a valuable resource for early-year learning. However, concerns exist regarding their potential negative impacts on children’s self-regulation, parent-child interaction and physical activity.

The primary aim of this study is to understand the longitudinal impact of IED use, in particular duration (hours per day) and mode (educational vs non-educational; age-appropriate vs non-age-appropriate), on emerging abilities (ie, self-regulation, social development, executive function, language and numeracy) in 3-year-old to 5-year-old children. The secondary aims are to explore the impact of IEDs on health-related outcomes (ie, body mass index and motor skills), behavioural outcomes (ie, movement behaviour, parent-child interaction) and educational outcomes (ie, school readiness).

**Methods and analysis:**

We aim to recruit 1377 children from economically diverse areas in the Mid and North of England, UK. We will measure children’s exposure to IEDs using a mobile sensing application tool which records app usage, while the primary outcome, emerging abilities, will be measured through the Early Years Toolbox. The secondary outcome measures will include the following: accelerometry (24-hour movement behaviour), National Institute of Health (NIH) Toolbox (motor skills), STIM-Q preschool questionnaire (parent-child interaction) and early years foundation stage profile (school readiness). We will employ multilevel regression models to examine the association between IED duration and mode with emerging abilities. We hope this study will contribute to the development of guidelines for parents and educators regarding the use of IEDs.

**Ethics and dissemination:**

The study has received approval from Sheffield Hallam University (ID: ER69550320). Engagement with the public and stakeholders will guide the dissemination plan. The insights gained from this project will be shared through publications and will inform policy briefs distributed to health and educational organisations.

**Trial registration number:**

NCT06810570.

**STRENGTHS AND LIMITATIONS OF THIS STUDY**

- We will gather a comprehensive array of developmental and health data to deepen our understanding of how interactive electronic devices (IEDs) influence the health and growth of young children.
- We will use direct measures to assess screen behaviour in children and validated, device-based measures to evaluate development, health and behavioural outcomes in young children.
- This observational study aims to assess the association between exposure to IEDs and developmental and health outcomes, while not addressing causality.
- Despite including a number of potential confounding variables, other confounders not included here might impact the association between the exposure and outcome variables, which introduces a moderate level of bias.

## Introduction

 Despite extensive research on the effects of television viewing on socio-emotional, cognitive and health outcomes in children,^1^,^3^ research on interactive electronic devices (IEDs), such as tablets and smartphones, is just beginning to emerge. IEDs are now commonplace in the lives of young children, with a high proportion of children aged 3 to 4 years in the UK going online on tablets (67%) or smartphones (43%) to play games and watch videos.^4^

Evidence-based recommendations for young children’s screen time are provided by the WHO and other international guidelines,^5^,^7^ which advise that children between the ages of 3 and 4 should limit screen time to no more than 1 hour per day.^8^ However, some argue there is a need for further evidence on the use of ‘contemporary screen technology’ (eg, tablets and smartphones) on health and developmental outcomes,^9^ with some considering that mobile devices could be less harmful regarding sedentary behaviour.^10^ There is also uncertainty between early years practitioners and policy makers regarding the benefits and harms of using tablets and smartphones. While they recognise the ease of access and interactive opportunities these devices offer, they also express concerns about the difficulties of parental supervision.^11^

The scientific evidence in this field is conflicting and tends to focus on older children, particularly teenagers.^12 13^ Evidence from a systematic review and meta-analysis shows that IEDs have a negative impact on sleep outcomes (inadequate sleep, poor sleep quality and excessive daytime sleepiness) in children and adolescents aged 6 to 19 years.^14^ Likewise, for young children (<6 years), there is cross-sectional evidence that IED usage is associated with shorter sleep and sleep disturbances.^15^ Furthermore, a systematic review linked smartphone overuse to visual impairment in children (>9 years) and young adults,^16^ while prolonged use (>5 hours per day) of tablets, smartphones, computers and video games combined was associated with obesity in adolescents.^17^ Excessive parental use of IEDs might also have a negative impact on children. A literature review reported that parental use of mobile devices while around young children is associated with fewer and more negative parent-child interactions, with parents being less sensitive and responsive to their children’s requests for attention.^18^

Conversely, it has been argued that interactive technology can enhance learning and communication^19^ by helping language learning in young children when content is co-viewed and discussed with parents.^20^ Similarly, a systematic review of the effect of tablets on learning found that most studies reported a positive impact on literacy development, mathematics, science, problem‐solving and self‐efficacy in young children (2 to 5 years). However, the review consisted primarily of observational studies, which reported teachers’ and parents’ opinions, and quality assessment was not conducted.^21^ Finally, some experts suggest that interactive screen technology can reduce the gap in learning between those from more affluent and deprived areas.^10^ For example, learning apps might enhance social and language skills for children living in poverty and otherwise disadvantaged environments, showing the potential to explore how these technologies could minimise the attainment gaps among children from diverse socioeconomic backgrounds.^10^ Other authors propose that using IEDs might not increase sedentary behaviour as much as other forms of passive screen viewing (eg, TV).^22 23^ They argue that some activity-based programmes in these devices might encourage imitation or participation^22^ or encourage children to explore the outdoors.^23^ However, there is a need for empirical research evidence to support these claims. Likewise, only a few studies have examined the association between IEDs and motor skills in young children, with contrasting results.^24 25^

Arguably, for young children, the most relevant outcome is socio-emotional and cognitive development. It is well known that behavioural and psychosocial experiences in early life can affect brain development and behaviour,^26^ and multiple factors can influence development at a young age, including maternal education, linguistic competence,^27^ parental psychopathology and socioeconomic status.^28^ However, social interactions and environments also have a significant influence.^29^ The use of IEDs, which is becoming increasingly frequent in young children’s lives, may have a broader impact that could affect developmental outcomes and have long-term effects. The evidence on the impact of IEDs on socio-emotional and self-regulatory skills is still contradictory,^30 31^ with researchers calling for more studies in this area.^19^ A recent narrative review reported experimental evidence that IEDs can be more beneficial for young children’s (0 to 5 years) learning and self-regulation than TV viewing. However, studies included in this review which examined the naturalist use of mobile devices show that an increase in use is associated with poorer language and self-regulation.^30^ Likewise, a longitudinal study also found that young children (3 to 5 years) with higher levels of programme viewing (TV or internet programmes on any device) had increased externalising behaviour problems and total psychological difficulties 12 months later. Additionally, children who used apps for more than 30 min per day had lower inhibitory control (resisting distractions and impulsive behaviours) 1 year later. However, the study only included a small sample of children (n=185), looked at multiple devices, including TV and video games, and did not explore the screen media content.^32^

### Patient and public involvement and engagement (PPIE)

In the PPIE that informed the present study, early years practitioners and public health consultants expressed their recognition of the potential of using IEDs as learning tools. However, they also voiced concerns about the long-term effects of these devices on children’s communication, social and physical skills and how children are impacted by their parents’ use of these devices.^11^ Similarly, parents acknowledged the educational benefits of IEDs but highlighted the negative impacts on sleep, self-regulation and physical activity. They admitted to using these devices for ‘babysitting’ and entertainment when they are busy but considered it important to manage their child’s use, as well as their own, as a positive role model.

Informed by the UK Standards for Public Involvement,^33^ we engaged parents and early years practitioners from the beginning of the project. We recruited 10 early years practitioners and nine parents, all of whom received training from our PPIE lead (CS). The PPIE groups will meet online or in person every 6 months. They have already contributed to developing all recruitment materials, including information sheets, informed consent forms, videos and the project website. Throughout the project, the research team will share findings with the PPIE groups and seek clarification of which findings resonate most with stakeholders like themselves. They will support the project’s dissemination strategy by revising plain language summaries and newsletters for distribution to parents and early years settings and co-present findings at public involvement activities, such as Knowledge Cafés.

### Aims

This manuscript describes the study protocol of the Investigating Kids’ Interactions with Digital Screens (iKids) study. To date, few studies have focused on the impact of IEDs on health and health-related behaviour outcomes, and those primarily use cross-sectional designs^34^,^36^ with inconsistent evidence on the benefits and harms. Therefore, the primary aim of this study is to explore the association between IED use, as measured by duration and mode (eg, age-appropriate/inappropriate, educational/non-educational) and emerging abilities including self-regulation, social development, executive function, language and numeracy. We also aim to explore the optimum dose (duration) that could enhance the positive effects of IED use on emerging abilities while minimising any negative effects. The secondary aim of this study is to explore the longitudinal association between IED use (duration and mode) and secondary outcomes (ie, body mass index (BMI) z-score, movement behaviour, motor skills, parent-child interaction and school readiness). Finally, using the socioecological model^37^ as a theoretical framework, we will investigate the correlates of IED use (duration and mode) at the individual (gender and ethnicity), interpersonal (maternal education and parenting style) and organisational (childcare policy and attendance) levels.

## Methods and analysis

This is a 1-year longitudinal study which will follow the Strengthening the Reporting of Observational Studies in Epidemiology statement.^38^ The study was registered at Clinical Trials (registration number NCT06810570) before participant recruitment started. Information about the study, including recruitment materials, information sheets and videos, is presented on the project website www.ikidsstudy.com. The project started in March 2025, with recruitment starting in November 2024. The project is expected to end in October 2027.

### Sampling and participant eligibility

We will use a cluster sampling approach to recruit school nurseries, day nurseries or childminders (educational unit clusters) located in the Mid and North of England. Children will be eligible to participate if they are between 36 and 48 months old at enrolment, have parent/carer consent for participation and have provided verbal assent. Children will be ineligible if parents or the child does not speak and/or understand English or if the child is clinically diagnosed with a developmental disorder by a medical professional prior to baseline.

### Sample size calculation

We base our estimates of sample size on estimates of the magnitude of the effect of IED duration (hours per day) and IED mode (educational vs non-educational; age-appropriate vs non-age-appropriate) on the primary outcome of emerging abilities measured as a composite score (ie, self-regulation, social development, executive function, language and numeracy) assessed by the Early Years Toolbox.^39^ An expected effect size of 0.01 was adopted based on the findings of the Kuzik *et al*^40^ study, which examined the association between meeting the Canadian screen time recommendation (no more than 1 h/day if 3 to 4 years old) and composite development score, including physical, cognitive and social-emotional development.

Calculations based on the primary outcome (ie, emerging abilities) are under the alternative assumptions of (1) a linear relationship between IED time and emerging abilities (following the methods of Kuzik *et al*^40^) and (2) a non-linear relationship with a functional form, allowing a turning point or plateauing effect within the range of the data, with the final sample size conservatively estimated to be the larger of these two figures.

A sample size of 695 (adjusted for clustering) before attrition loss would achieve 80% power to detect an effect on the emerging abilities score at a significance level of 0.05 for the given effect size (0.01). The attrition loss was informed by a 12-month longitudinal study which investigated the associations between electronic application use and media programme viewing with cognitive and psychosocial development in preschool children aged 4–5 years,^32^ which reported 52% attrition from recruitment and a further 21.3% attrition between baseline and 12-month follow-up in this study. We anticipate lower rates of missing data in our study, as the exposure (IED use) will be measured by an app rather than a parent questionnaire. We will also undertake subsequent visits to the educational units to recruit children absent at the initial visit and will offer incentives to encourage completion. Therefore, we are assuming 40% attrition pre-baseline and 15% attrition between baseline and follow-up, resulting in an estimated sample of 1377 children to be screened for eligibility. Sample size calculations were conducted using Power Analysis Software (PASS) 2022.^41^

### Recruitment

We expect to recruit an average of 13 children per school nursery or day nursery and an average of 1 child per childminder, recruiting 51 educational units of each type (ie, school nurseries, day nurseries and childminders) or 153 educational units in total. We aim to recruit educational units located in the Mid and North areas of England, including the county areas of West Yorkshire, South Yorkshire, Derbyshire and Nottinghamshire. We will make an effort to recruit educational units evenly across the index of multiple deprivations (IMD)^42^ tertiles in each Council.

We will employ several strategies for recruitment. First, we will seek the assistance of Early Years Public Health consultants who work in these areas to act as gatekeepers for the recruitment of educational units. We will also send study flyers by post to these educational units and advertise in the government’s early years pages, newsletters, social media and specific networks for early years settings (eg, Nursery World). In the second stage, we will target parents directly by promoting the study through social media communities and government organisations tailored to parents of young children.

When recruiting from educational units, headteachers, nursery managers and childminders will receive information about the study via email. Once they have signed the informed consent, we will visit these settings to promote the iKids study using posters and flyers. Parents of children who meet the age criteria (36 to 48 months) will receive a study information package detailing how to enrol. Each educational unit will receive an incentive of £100 at each time point of data collection, while parents or carers will receive a gift card of £30 at each data collection time point (baseline and follow-up).

### Measurements

Data collection will take place when children are aged between 36 and 48 months (‘baseline’) and 1 year later when children are 48 to 60 months (‘follow-up’). Apart from school readiness, which will only be measured at follow-up, all other variables will be measured at both baseline and follow-up. Table 1 provides an overview of the exposure, outcome measures and timeline for data collection.

**Table 1:** Exposure, outcomes and control variables description and timescale

Variable	Description	Timescale
Exposure		
Interactive electronic devices	Screen time and app usage	Baseline and follow-up
Primary Outcome		
Emerging abilities	Composite and domain scores of the following: (1) visual-spatial working memory, (2) phonological working memory, (3) inhibition, (4) shifting, (5) numeracy and mathematical concepts, (6) expressive vocabulary, (7) self-regulation and social development	Baseline and follow-up
Secondary outcomes		
BMI	BMI age and sex normed z-score	Baseline and follow-up
24-hour movement behaviour	Compositional and per behaviour analysis: (1) sleep, (2) sedentary behaviour, (3) physical activity.	Baseline and follow-up
Motor skills	Motor skills z-score and per skills: (1) upper extremity strength, (2) balance, (3) explosive leg power, (4) functional mobility Fine motor skills: (1) dexterity	Baseline and follow-up
Parent-child interaction	Cognitive home environment	Baseline and follow-up
School readiness	Total school readiness and per areas of learning: (1) communication and language, (2) physical development, (3) personal, social and emotional development, (4) literacy and mathematics	Follow-up
Control variables		
Participant demographics	Sex, age, ethnicity	Baseline
Community ethnicity congruence	Proportion of residents in the child’s postcode region who are the same ethnicity as the child	Baseline and follow-up
Other screen viewing (time and contextual)	Time spent on other screen activities, including television and video games Contextual information regarding screen viewing, including when and where it occurs and parents’ attitudes towards their own screen usage when they are around their children	Baseline and follow-up
Caregiver education	Highest level of caregiver education completed by a member of the child’s household	Baseline and follow-up
Parenting style	Parenting styles categorising as follows: (1) authoritative, (2) authoritarian, or (3) permissive	Baseline and follow-up
Hours of childcare attendance	The number of hours per week that the child attends childcare, as reported by the setting manager	Baseline and follow-up
Screen viewing policy	Presence of a policy or general practice that pertains specifically to the amount of time children can watch or play or work on a tablet at the early years setting	Baseline and follow-up
Parent smartphone addiction score	Caregiver smartphone dependency	Baseline and follow-up
Unit type	Type of setting the child attends: (1) school nursery, (2) daily nursery and (3) childminder	Baseline and follow-up

BMI, body mass index.

### Exposure measures

Parents of eligible children who agree to participate will need to provide information about the type of IED their child has access to. The devices include mobile phones and tablets that run on the following operating systems: Android, iOS and Fire OS. We will also inquire if the child shares the device with another family member when they register in the study.

Parents will be asked to download the Effortless Assessment of Risk States (EARS) app from either the Google Play Store or the App Store, or via an APK installer on the mobile device or tablet that their child uses. This app, developed by Ksana Health,^43^ includes various features such as language capture, semantic categorisation, geolocation, and music or mood profiles. The app was customised for this study to only collect data on screen time and app usage. While this app has previously been used in mental health studies,^44 45^ it has not yet been employed to measure screen time in young children. The need for improved methodology in assessing screen time has been recognised as a challenge in a global Delphi study aimed at enhancing the surveillance of movement behaviour.^46^ The use of an app rather than the usual self-report questionnaire^47^ might help to inform the methodology of other studies in the field.

Participants will receive instructions on downloading the app and scanning a unique participant Quick Response (QR) code on their device, allowing the data to remain confidential. The EARS app will run in the background of the IED for 7 days and be continuously uploaded and downloaded to a cloud server.

Families of participants who share a device will be required to download the app and maintain a diary documenting the times the device is given to and taken from the child. At the time of data analysis, we will use the information from the diary to extract data that corresponds specifically to the child’s usage period.

Data recorded from the EARS app will be processed as IED duration or IED mode as follows:

1. IED duration: we will add the total time in minutes of use of the IED device by the child across 7 days and calculate the average time in minutes per day per child. If the child does not use IEDs, the time will be recorded as zero.
2. IED mode: we will record the app classifications based on the category provided by the App Store or Google Play. Two researchers will then group these apps in the following categories: (a) educational apps; (b) game apps age-appropriate (app is appropriate for 3+ years old); (c) game apps non-age-appropriate (app appropriate for 5+ years old); (d) streaming videos age-appropriate (ie, video streaming services set to the age restriction of 5 or less); (e) streaming videos non-age-appropriate; (f) others (eg, video chats, photos, maps). Discrepancies between researchers will be resolved through discussion. To assess interrater agreement for coding the app categories, we will use Cohen’s kappa.

The mode of IED categorisation will be further combined into time spent: (1) educational (app educational) versus non-educational (all others), (2) entertainment age-appropriate (educational apps, games apps and streaming videos age-appropriate) versus entertainment non-age-appropriate (game apps and streaming videos non-age-appropriate).

### Primary outcome

The primary outcome is a composite score of the emerging abilities measured by the Early Years Toolbox, which has been previously validated for this age group.^39^ Data will be recorded on an iPad at the educational unit which the child attends. Emerging abilities assessment and Early Years Toolbox task include the following: (1) visual-spatial working memory—‘Mr Ant’ task, (2) phonological working memory—‘Not this’ task, (3) inhibition is the ability to control behaviours—‘Go/No-Go’ task, (4) shifting—‘Card Sorting’ task, (5) numeracy and mathematical concepts—‘Early Numeracy’ task, (6) expressive vocabulary—‘Vocab’ task, (7) self-regulation and social development—34-item questionnaire ‘Child Self-Regulation & Behaviour Questionnaire’, which will be answered by an early years practitioner at the educational unit.

A z-score will be calculated for each of the above developmental outcome variables, and the mean z-score for each emerging ability outcome will be used to create a composite score.

### Secondary outcomes

The set of secondary outcomes to be considered is based on those analysed in the SUNRISE study protocol, International Study of Movement Behaviours in the Early Years,^48^ which currently includes participants from 64 countries and provides evidence of the feasibility of the measures in this age group.^49^,^51^

The secondary outcome measures considered in the current analysis are as follows:

1. BMI z-score, calculated according to the BMI reference curves for the UK.^52^
2. 24-hour movement behaviour, assessed by waist-worn ActiGraph GT3X-BT accelerometers. Children will be advised to wear the accelerometer for 1 week to obtain a minimum of 3 days of at least 16 hours of recorded data.^53^ We will follow the Sleep and Activity Database for the Early Years (SADEY) data harmonisation protocol for preschoolers^54^ for processing the data.
3. Total motor development score, assessed by the National Institute of Health (NIH) Toolbox^55^ and according to the protocols advised by the Motor Domain Group for this age group.^56^ We will test four gross motor skills tests (ie, ‘Standing long jump’, ‘Supine-timed up and go’, ‘One-legged standing balance’ and ‘handgrip dynamometer’) and one fine motor skills (ie, 9-hole pegboard test). We will report individual scores, calculate a z-score for each individual task and combine them to obtain the total score.
4. Parent-child interaction, measured using the StimQ preschool questionnaire,^57^ will be administered over the phone through a parent/carer interview. Total scores will be calculated by summing up the subscale scores (ie, reading, parental involvement in developmental advance, parental verbal responsivity and availability of learning materials).
5. School readiness will be measured by the Early Years’ Foundation Stage Profile (EYFSP).^58^ The EYFSP will be provided by the educational unit before children start formal school (Year 1). We will calculate the child’s total school readiness score (range 12–24) by adding each learning goal score for the prime areas of learning (communication and language, physical development, personal, social and emotional development) and two of the specific areas of learning (literacy and mathematics).

### Controlling variables

We will include the following control variables in the analyses: (1) sex; (2) age; (3) ethnicity; (4) community ethnicity congruence (ie, proportion of residents in the child’s postcode region who are the same ethnicity as the child); (5) other screen viewing (time and contextual); (6) caregiver education; (7) parenting style; (8) hours of childcare attendance; (9) presence of screen viewing policy at the educational unit; (10) parent smartphone addiction score; (11) child baseline emerging ability score; (12) unit type: school nursery, daily nursery and childminder.

### Data cleaning and assessment of missing data

We will assess whether the values of continuous variables are within range, the plausibility of means and SD, and the validity of coded categories. We will assess outliers from graphical methods and inspect leverage/Mahalanobis distances, discrepancies and influence statistics.

For small proportions of missing data (below 5% of the totality of the data), which appear to be missing at random or completely at random (assessed by Little’s χ^2^ test and/or separate variance t-tests), we will consider complete case analyses. If the amount or pattern of missing data precludes complete case analysis, we will consider multiple imputations. If imputation is conducted, we will conduct a sensitivity analysis by comparing results derived from data with and without imputation.

### Analysis

Continuous and categorical outcomes will be summarised descriptively. We will assess the need for variable transformations to stabilise variance or achieve normality.

### Inferential analysis

The following analysis will be conducted:

#### Multilevel regression modelling of primary outcome

We conceptualise a two-level random intercepts multilevel model, with children clustered within educational units at the first instance, and an alternative model with children clustered within families.

This model, in which we consider emerging abilities at follow-up to be the outcome measure, is designed to answer the primary aim: the association between IEDs and emerging abilities. We will assess the variance partition coefficient (VPC) via a null model before proceeding to a covariate model. We will consider merging the levels in the model if VPC statistics reveal negligible clustering effects (negligible residual variance at the level of the educational unit). We anticipate the variance between children within a family will be very small, and there will be a small number of clusters within families, considering the narrow age range of children (36 to 48 months). Therefore, this cluster effect in the model in which children are considered to be clustered within families might be disregarded; nevertheless, we will conduct a sensitivity analysis, considering a single-level (child) model to confirm data hierarchy.

We will conduct non-linear multiple regression modelling if there is evidence for a non-linear relationship between the level of IED usage at baseline and emerging abilities at follow-up, allowing for a single maximum (corresponding to optimum levels of baseline IED usage) or plateau; otherwise, we will conduct linear regression models. If data indicate an optimum IED level of usage associated with a specific maximum value of emerging abilities score at follow-up, we will consider alternative non-linear functional forms with maxima or plateauing features, including polynomial (eg, quadratic) and logarithmic forms and piecewise functions. We will compare the fit of multiple distributions in the vicinity of any turning point and select the best-fitting model in this region to maximise the accuracy with which the maximum value of emerging abilities may be obtained. We will fit confidence intervals around the function to derive a range of values for the maximum value.

We will conduct both non-fully adjusted and adjusted models, with adjusted models adjusted for all covariates at each level of the model. Non-adjusted models will include (1) the single determinant of the level of IED usage (duration) at baseline, (2) the determinants of IED predominant mode at baseline (as defined above) and (3) determinants of IED duration and mode. These determinants will all be added at the *child* level of the model. To capture any differential effects in assessing levels of IED usage with differing predominant modes of use, we will include first-order interactions within unadjusted models. Any interaction revealed to be of substantive importance will be retained in a re-cast model alongside all main effects. Adjusted models will be based on the included variables of both IED duration and IED predominant mode, any interactions of substantive importance, and all-controlling covariates at the appropriate level of the model. We will retain all main effects in the adjusted model, assess collinearity in adjusted models and consider deletion of controlling covariates if excessive collinearity is apparent (variance inflation factor ≥5 for any variable).

#### Multilevel regression modelling of secondary outcomes

We will conduct the same model structure as for the main analyses of the primary outcome. If any evidence is revealed for non-linearity between the level of IED duration and the secondary outcomes, we will consider non-linear modelling. We will adjust for the same set of covariates and interactions defined for the primary outcome.

#### Multilevel regression modelling: subsidiary analyses

To explore the socioecological correlates of IED duration at the individual, interpersonal and organisational levels, we will conduct random intercepts and multiple linear regression modelling on the outcome of IED duration at baseline. A two-level hierarchical structure will be used, with variables designated as individual or interpersonal attached at the lower (child) level and variables designated as organisational attached at the upper (educational unit) level. Any relationships revealed during this process will be used in future modelling to generate hypotheses within a wider structural equation modelling framework.

#### Sensitivity analysis and data reporting

For the primary outcome, we plan to conduct sensitivity analyses to assess the sensitivity of the model to certain assumptions. We will compare parameter estimates of tested variables in unadjusted and adjusted models. For the multilevel modelling of the relationship between IED duration and emerging abilities, we will conduct both random slopes and random intercepts models and assess variation in slopes between higher-level units. If data imputation is viable, we will run models with and without imputed data.

For linear and non-linear regression models of continuous numerical outcomes, in the main and subsidiary analyses, we will report all unstandardised parameter estimates with associated 95% CIs and p values. For logistic regression models, we will report all ORs with associated 95% CIs and p values. If evidence is revealed for a non-linear trend, we will report the functional form of the best-fitting curve and identify the location of any maximum or commencement of plateauing effects. We will check all regression modelling assumptions, including homogeneity of variance and normality of outcome variables for each value of an independent variable, using residual analysis. Statistical analyses will be conducted using MLwiN V.3.06^59^ and Stata V.17.^60^

## Discussion

The impact of IEDs on children and young people has gained ongoing attention from the media,^61 62^ policy makers^63 64^ and the scientific community.^65 66^ Parents and educators increasingly seek effective interventions to help guide children’s use of these devices.^67^ However, the scientific evidence surrounding this issue is still conflicting and has primarily been obtained from cross-sectional studies.^34^,^36^ Additionally, there is a need to establish a potential dose-response relationship between IED use and health outcomes,^68^ which is crucial for shaping these guidelines.

Guided by the Behavioural Epidemiology Framework,^69^ the associations between IED use and development and health outcomes, including dose-response relationships, should first be established to inform population health guidelines and before intervention development. The iKids study aims to enhance our understanding of both the benefits and harms associated with the use of IEDs. Specifically, it will explore the dose-response relationship and its impact on development and health outcomes. The findings from this study will provide valuable evidence to guide future health guidelines and the development of intervention strategies.

## Ethics and dissemination

The study will comply with the Economic and Social Research Council’s research ethics framework. Ethical approval was sought for all aspects of the work from Sheffield Hallam University (ID: ER69550320). All researchers in the project will work in accordance with the educational unit safeguarding policies. We will request informed written parental/carer consent while children give their verbal assent to participate. Early years practitioners involved in the project will also be asked for their written consent.

Discussions with the public and stakeholder engagement groups will inform the dissemination plan. The learning from this project will be disseminated through publications and will inform policy briefs distributed to health and educational organisations. We will also offer Knowledge Café events and information (newsletters, website) to early years settings.
