# Supplementary material for: iKids study protocol: a longitudinal study to understand the impact of interactive electronic devices on the development and health of young children in England
Source: BMJ Open. 2025 Aug 1;15(7):e101523. doi: 10.1136/bmjopen-2025-101523 (PMC12314959; doi:10.1136/bmjopen-2025-101523)
Supplement: Supplementary file 1 [file bmjopen-2025-101523.reviewer_comments.pdf]

PEER REVIEW HISTORY

BMJ Open publishes all reviews undertaken for accepted manuscripts. Reviewers are asked to complete a checklist review form and are provided with free text boxes to elaborate on their assessment. These free text comments are reproduced below.

ARTICLE DETAILS

Title (Provisional)

iKids study protocol: A longitudinal study to understand the impact of interactive electronic devices on the development and health of young children in England

Authors

De Azevedo, Liane Beretta; Stephenson, J; Hughes, Amy; Retzler, Jenny; Reilly, John J; Fairclough, Stuart; Okely, Tony; Jones, Daniel; Smith, Christine; Greca, João Paulo de Aguiar; Marr, Colette

---

VERSION 1 - REVIEW

**Reviewer**	**1**
**Name**	**Elgier, Angel Manuel**
**Affiliation**	**Universidad de Buenos Aires**
**Date**	**21-Apr-2025**
**COI**	**None**

The manuscript is well-written, and the rationale for the iKids study is compelling. The proposed methodology is comprehensive, combining objective measurements (e.g., mobile sensing app, accelerometry, validated developmental assessments) with an appropriate analytical framework (multilevel modeling with sensitivity analyses). The study's potential to inform public health and educational guidelines is noteworthy.

I have only a few minor suggestions for clarification:

Clarify data handling for shared devices: The protocol mentions a diary to track when the child is using the device, but further details would be helpful to assess how accurate exposure data will be ensured in these cases.

App categorization: The method for classifying app content (educational vs. non-educational; age-appropriate vs. not) is sound but could benefit from more explanation on how inter-rater agreement will be managed, especially if classification is done manually.

Statistical approach: While the modeling strategy is thorough, consider briefly noting how potential clustering at the family level (e.g., siblings in the same household) will be handled, if applicable.

Data sharing: It might be useful to add a brief statement on whether de-identified data will be made available to external researchers upon request, in addition to the mention of SHURDA.

Overall, this is a well-conceived and methodologically rigorous protocol. I look forward to seeing the results of this study in the future.

**Reviewer**	**2**
**Name**	**Mezawa, Hidetoshi**
**Affiliation**	**National Center for Child Health and Development**
**Date**	**18-Jun-2025**
**COI**	**None**

Thank you for the opportunity to review this valuable study protocol. In modern society, media exposure is an unavoidable aspect of daily life, and the modes of exposure to media content have changed significantly in recent years. As such, strategies to minimize its negative impact must be continuously updated. Conducting a new cohort study to address these issues is therefore highly meaningful. I respectfully suggest that the following points be addressed in the study protocol:

1. Please include a detailed table outlining the specific types of information to be collected and the corresponding timeline.

2. Although the EARS app is planned for exposure assessment, please clarify how media exposure through devices other than some installed devices will be captured.

3. Please specify how the study will evaluate participants with no media exposure or those who do not use the app.

4. The recruitment strategy based on geographic sampling appears appropriate; however, please provide justification for how this approach ensures representativeness of media exposure variance.

5. In early childhood, parental attitudes toward media and parents' own media use are important confounding factors. While the study includes the smartphone addiction score, it appears that media exposure among otherwise healthy users may not be fully captured. Please clarify whether there is a plan to collect this information.

VERSION 1 - AUTHOR RESPONSE

Reviewer 1 comments

Comments	Response
The manuscript is well-written, and the rationale for the iKids study is compelling. The proposed methodology is comprehensive, combining objective measurements (e.g., mobile sensing app, accelerometery, validated developmental assessments) with an appropriate analytical framework (multilevel modelling with sensitivity analyses). The study's potential to inform public health and educational guidelines is noteworthy. I have only a few minor suggestions for clarification.	We sincerely appreciate the reviewer's thoughtful compliment and encouragement.
Clarify data handling for shared devices: The protocol mentions a diary to track when the child is using the device, but further details would be helpful to assess how accurate exposure data will be ensured in these cases.	Capturing data from devices shared among family members can be challenging, and there is no simple solution. We provided a few extra points for clarification, including that parents will need to download the app and provide information about the time their child used it. We will then extract the data collected during that reported time period for analysis.
App categorization: The method for classifying app content (educational vs. non-educational; age-appropriate vs. not) is sound but could benefit from more explanation on how inter-rater agreement will be managed, especially if classification is done manually.	Although App Store labels provide information on app classification and age appropriateness. We will create an app categorization scheme table in which apps would fit in each of the categories listed in the manuscript. Two researchers will classify these apps, and inter-rater agreement will be assessed using Cohen's Kappa. Further details were added in the manuscript.
Statistical approach: While the modeling strategy is thorough, consider briefly noting how potential clustering at the family level (e.g., siblings in the same household) will be handled, if applicable.	This analysis will account for clustering by using multilevel models, where children are considered at the lower level and are nested within households at the upper level. Since we are focusing on a narrow age range at baseline, specifically children aged 36 to 48 months, we expect the clustering effect to be minimal, primarily involving twin children. To assess this, we will first calculate the proportion of variance in the outcome attributed to two components: (i) variance between families and (ii)

	variance between children within families. The variance partition coefficient, which represents the variance between families as a proportion of the total variance, will then be computed. If this coefficient is very small, we may disregard the clustering effect. However, as a precaution, we will conduct a sensitivity analysis both with and without the data hierarchy to ensure that the results are not affected by clustering within families. This information has been synthesized and incorporated into the manuscript.
Data sharing: It might be useful to add a brief statement on whether de-identified data will be made available to external researchers upon request, in addition to the mention of SHURDA.	We added the information that after **a 6-month** embargo, de-identified data will be deposited in a community-recognised repository under a CC-BY license, as guided by the funder (i.e. NIHR).

Reviewer 2

Comments	Response
Thank you for the opportunity to review this valuable study protocol. In modern society, media exposure is an unavoidable aspect of daily life, and the modes of exposure to media content have changed significantly in recent years. As such, strategies to minimize its negative impact must be continuously updated. Conducting a new cohort study to address these issues is therefore highly meaningful. I respectfully suggest that the following points be addressed in the study protocol.	We acknowledge the significance and relevance of this study, and we appreciate your comments.
Please include a detailed table outlining the specific types of information to be	We have now included a table where we outline the exposure, outcome measures, and covariates along with the timeline for data collection.

collected and the corresponding timeline.	
Although the EARS app is planned for exposure assessment, please clarify how media exposure through devices other than the installed devices will be captured.	The questionnaires we are using are informed by the SUNRISE study protocol (questionnaires are reported on the supplement files of Okely et al., 2021, https://doi.org/10.1136/bmjopen-2021-049267). While these measurements were not detailed in the first submitted version of the protocol, they are now briefly explained and listed in the controlling variables and in Table 1.<br><br>The questionnaire will specifically gather information about various screen time activities, including television and video games. It will include questions such as: "How much time did the child participating in this study spend using electronic screen devices, such as a smartphone, tablet, video game, or watching television or videos on the internet?" Additionally, the questionnaire will collect contextual information, such as device usage before bedtime, the presence of screens in the bedroom, and whether parents use devices while in the presence of the child.
Please specify how the study will evaluate participants with no media exposure or those who do not use the app.	Children who do not use tablets or smartphones will still be included in the study. For these children, the time spent on these devices will be recorded as zero. This information has now been incorporated into the manuscript.
The recruitment strategy based on geographic sampling appears appropriate; however, please provide justification for how this approach ensures representativeness of media exposure variance	In our study, we stratified the geographical representation based on the Index of Multiple Deprivation (IMD), which is the official measure of relative deprivation in England. This measure takes into account several domains, such as Income, Employment, Education, Health, Crime, Barriers to Housing and Services, and the Living Environment (Department for Communities and Local Government, Health Equity Evidence Centre, 2024; https://www.heec.co.uk/resource/understanding-the-index-of-multiple-deprivation-imd-in-public-health-research/). Consequently, we stratified our sample according to sociodemographic status.<br><br>The existing evidence regarding the association between the use of interactive electronic devices and socioeconomic position is inconsistent. Some studies found no significant association (Maata et al., 2017, http://dx.doi.org/10.1136/bmjopen-2017-

	016690; Park et al., 2018, https://doi.org/10.3346/jkms.2018.33.e219; Pempek et al., 2016, DOI 10.1007/s10826-016-0413-x), while others reported a negative correlation (Bui et al., 2022 https://doi.org/10.1007/s10826-021-02126-8; Kardas et al., 2023 http://dx.doi.org/10.5546/aap.2023-10122.eng; Rodrigues et al., 2020 https://doi.org/10.1186/s12889-020-09026-4; Shawcroft, 2023, https://orcid.org/0000-0002-6502-6805). As this is a new area of research, we lack knowledge of other population characteristics that could give us media exposure representativeness. Thus, using the IMD approach appears to be the most appropriate method for stratifying our sample, other than helping us to explore whether economic inequalities contribute to interactive electronic device variance.<br><br>On the other hand, there is consistent evidence demonstrating a negative association between socioeconomic status and early childhood development, which is our primary outcome (Bradley, Letourneau, N. L., et al., 2013; https://doi.org/10.1177/1063426611421007; https://doi.org/10.1111/jcpp.14082). Therefore, recruitment based on sociodemographic representativeness seems to be the best approach.
In early childhood, parental attitudes toward media and parents' own media use are important confounding factors. While the study includes the smartphone addiction score, it appears that media exposure among otherwise healthy users may not be fully captured. Please clarify whether there is a plan to collect this information.	There is evidence of a positive correlation between the Smartphone Addiction Scale-Short Version (SAS-SV, Kwon et al., 2013), which we are using in our study, and smartphone use (Hamamura et al., 2023 https://doi.org/10.1186/s40359-023-01095-5; Vaghasiya et al., 2023 DOI: 10.4103/ipj.ipj_53_23). Therefore, even though we are not directly measuring parental smartphone usage, we can infer that parents who are less dependent on their smartphones (i.e., healthier users) may be more likely to have lower SAS-SV scores.<br><br>Additionally, the parent-reported questionnaire includes questions about parents' attitudes towards their own screen usage when they are with their children, such as during family meals, while busy, or when going for a walk. Based on this, we believe we have sufficient information about parents' attitudes toward these devices and prefer not to increase the

	participants' burden by adding another measurement.
